# Maccaroni

**Published:** 1877-11

**Authors:** 


### MACCARONI

Is an article of food which we should like to see in more common use in this country. When our people learn to make it, as well as they make it in Naples; and what is equally important to cook it as the Neapolitans do, it will be as much used here as it is there, for it is, or might be, cheap and healthful.

The flour is specially ground for it, and the best article of maccaroni is retailed in the Neapolitan provinces for five and six cents a pound. A commoner and coarser article is much used by the common people, which sells for about half this price. It is difficult to imagine what the *basse gente,* the lower classes, would do without maccaroni.

Here, we think, it is usually baked with cheese; a style in which no Italian could be induced to eat it. They regard baked cheese as very indigestible. They boil their maccaroni until it is tender, which ordinarily requires about ten minutes- and then serve it up with butter or the sauce of the *ragout.* To make this dish they take a piece of beef without bone, and after cutting an onion into small pieces and cooking it thoroughly in a kettle, they place the meat on the onion, and after it is partially cooked they add tomatoes, prepared as they would be for stewing; adding more from time to time and sometimes water and cooking for three or four hours. The meat is then served up by itself and the tomato sauce is poured over the maccaroni. They always have some grated cheese on the table to be sprinkled on the maccaroni by those who like it.

Twice a week, Sundays and Thursdays the year round, this forms the dinner for four-fifths of a population of eight millions of people. It is both healthful and inexpensive.

